# Chemical Composition Assessment of Structural Parts (Seeds, Peel, Pulp) of *Physalis alkekengi* L. Fruits

**DOI:** 10.3390/molecules27185787

**Published:** 2022-09-07

**Authors:** Venelina Popova, Zhana Petkova, Nadezhda Mazova, Tanya Ivanova, Nadezhda Petkova, Magdalena Stoyanova, Albena Stoyanova, Sezai Ercisli, Zuhal Okcu, Sona Skrovankova, Jiri Mlcek

**Affiliations:** 1 Department of Tobacco, Sugar, Vegetable and Essential Oils, University of Food Technologies, 4002 Plovdiv, Bulgaria; 2 Department of Chemical Technology, Faculty of Chemistry, University of Plovdiv “Paisii Hilendarski”, 4000 Plovdiv, Bulgaria; 3 Department of Engineering Ecology, University of Food Technologies, 4002 Plovdiv, Bulgaria; 4 Department of Organic Chemistry and Inorganic Chemistry, University of Food Technologies, 4002 Plovdiv, Bulgaria; 5 Department of Analytical Chemistry and Physical Chemistry, University of Food Technologies, 4002 Plovdiv, Bulgaria; 6 Department of Horticulture, Atatürk University, 25240 Erzurum, Turkey; 7 Department of Gastronomy, Faculty of Tourism, Ataturk University, 25240 Erzurum, Turkey; 8 Department of Food Analysis and Chemistry, Tomas Bata University in Zlin, 76001 Zlin, Czech Republic

**Keywords:** *Physalis alkekengi*, bladder cherry fruit, seeds, peel, pulp, oil, composition, bioactive compounds, concretes

## Abstract

In recent years there has been an extensive search for nature-based products with functional potential. All structural parts of *Physalis alkekengi* (bladder cherry), including fruits, pulp, and less-explored parts, such as seeds and peel, can be considered sources of functional macro- and micronutrients, bioactive compounds, such as vitamins, minerals, polyphenols, and polyunsaturated fatty acids, and dietetic fiber. The chemical composition of all fruit structural parts (seeds, peel, and pulp) of two phenotypes of *P. alkekengi* were studied. The seeds were found to be a rich source of oil, yielding 14–17%, with abundant amounts of unsaturated fatty acids (over 88%) and tocopherols, or vitamin E (up to 5378 mg/kg dw; dry weight). The predominant fatty acid in the seed oils was linoleic acid, followed by oleic acid. The seeds contained most of the fruit’s protein (16–19% dw) and fiber (6–8% dw). The peel oil differed significantly from the seed oil in fatty acid and tocopherol composition. Seed cakes, the waste after oil extraction, contained arginine and aspartic acid as the main amino acids; valine, phenylalanine, threonine, and isoleucine were present in slightly higher amounts than the other essential amino acids. They were also rich in key minerals, such as K, Mg, Fe, and Zn. From the peel and pulp fractions were extracted fruit concretes, aromatic products with specific fragrance profiles, of which volatile compositions (GC-MS) were identified. The major volatiles in peel and pulp concretes were β-linalool, α-pinene, and γ-terpinene. The results from the investigation substantiated the potential of all the studied fruit structures as new sources of bioactive compounds that could be used as prospective sources in human and animal nutrition, while the aroma-active compounds in the concretes supported the plant’s potential in perfumery and cosmetics.

## 1. Introduction

*Physalis alkekengi* L. (family Solanaceae), also known as the Chinese lantern, Japanese lantern, bladder cherry, winter cherry, and by many other common names, is a species indigenous to Asia and Southern Europe, further naturalized worldwide [1,2]. Nowadays, the Chinese lantern is encountered as cultivated and ornamental varieties or as a wild-growing plant in various climatic zones, from Central and Southern Europe to South and Northeast Asia.

The species is the only one in the genus *Physalis*, which is found as wild populations in Bulgaria, growing in different regions at altitudes up to 1200–1500 m [3,4]. The local name of the species is ‘mekhunka’, and its preservation and use are regulated by the Medicinal Plants Act [5]. Survey data [6] have documented a steady export of about 760 kg fresh *P. alkekengi* fruit per year in the period 2001–2005. In Bulgaria, the Chinese lantern grows as a perennial plant with widely spreading roots and a slightly branched or unbranched stem with a height between 40 and 60 cm. The fruit ripen in August–September, presenting as small, oval, brightly colored berries containing numerous tiny seeds, and completely covered by the characteristic wide orange-red papery calyx (husk).

Despite its identification as a medicinal plant, there are practically no data from investigations of *P. alkekengi* phytochemical composition in Bulgaria, nor from studies of its biological activities or range of application. Ivanov et al. [7] reported that the fruits contain red pigments, physalin, citric, malic and tartaric acids, vitamin C, and bitter substances. The seeds alone can contain up to 25% of the oil [7]. According to numerous studies on the species in its natural areas of distribution, over 100 bioactive metabolites have been identified in the fruit and other aerial parts of the plant, including alkaloids, nucleosides, peptides, terpenoids, megastigmanes, aliphatic derivatives, organic acids, coumarins, sucrose esters, polysaccharides, and carotenoid derivatives [8,9,10,11,12,13,14,15,16,17].

The Chinese lantern has been recognized for centuries as a medicinal plant in the traditional medical practices of many countries, due to its anti-inflammatory, antibacterial, antiseptic, sedative, laxative, diuretic, hypoglycemic, spasmolytic, and other effects, as well as for the relief of malaria and syphilis symptoms [1,18,19]. In Chinese medicine, *P. alkekengi* (Physalis calyx seu Fructus) is a remedy for a number of diseases—from sore throat, eczemas, and rheumatism to hepatitis, urinary disorders, and tumors [1,2,10,14,18]. In turn, Bulgarian folk medicine recommends the use of fresh or dried fruit for the treatment of liver diseases, combining hepatitis and ascites [3]. Dried fruits are also used as a painkiller for kidney and bladder stones, inflammations of the urinary tract, and hemorrhoids. In topical application, fresh juice or whole fruits relieve skin irritations, wounds, and inflammation. A daily consumption of at least 10–15 fresh berries (or the equivalent 20 mL freshly squeezed juice) is highly recommended [3].

*P. alkekengi* is also recognized as a functional food, being a rich source of valuable nutrients—vitamins A and C, minerals, unsaturated fatty acids, phenolics, phytosterols, and pectic substances [18,20,21,22]. It should be noted that only fully ripened berries are suitable for consumption (unripe fruit and all aerial parts of the plant are toxic if swallowed), having a juicy texture, fresh flavor, and a slightly bitter taste, which normally disappears after fruit freezing.

There is relatively limited information about the cosmetic uses of *P. alkekengi* in the form of aqueous, ethanolic, and other extracts from the fruit and calyces, which are incorporated in different cosmetic formulations, taking advantage of its beneficial effects on the skin (protective, soothing, anti-ageing, anti-pigmentation, and other effects) [1,23]. Those and related investigations have supported the inclusion of *P. alkekengi* fruit and calyx extracts (CAS No 90082-67-0) in the Cosmetic Ingredient Database (CosIng) of the European Commission, in the category of cosmetic ingredients with skin conditioning functions [24].

To the best of our knowledge, there are not enough data on the distribution of phytonutrients and other chemical compounds among the structural parts of the fruit (peel, pulp, and seeds), nor on the characteristics of *P. alkekengi* growing in different regions of Bulgaria. The main objective of this study was therefore to provide a comparative assessment of the chemical composition of the structural parts of the fruit in two Bulgarian phenotypes of *Physalis alkekengi* L., thus supplementing the already existing knowledge about the species and expanding its possible prospective use in nutrition and cosmetics.

## 2. Materials and Methods

### 2.1. Plant Material

Two phenotypes of *Physalis alkekengi* found in Bulgaria were analyzed in this study. Fully ripe fruits (about 150 pieces for each phenotype) were collected in August—September 2020 from wild plant populations in Central Southern Bulgaria (PA-SB phenotype; the city of Plovdiv; 42°14’26” N 24°70’24” E), and North-Eastern Bulgaria (PA-NB phenotype; near Ivanski village, Shumen region; 43°07’24” N 27°04’35” E). Species identification was confirmed by the botanist at the Department of Botany, Plovdiv University, Bulgaria.

**Figure 1:**
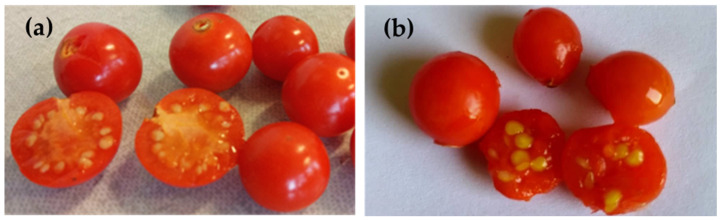
Fresh fruits of *P. alkekengi*: (**a**) PA-SB phenotype from Central Southern Bulgaria; (**b**) PA-NB phenotype from North-Eastern Bulgaria. Photos by authors.

According to the objectives of the study, fresh fruits (Figure 1) were de-husked and carefully divided into structural parts (seeds, peel, and pulp), that were analyzed individually or in mixture due to determination requirements.

### 2.2. Basic Evaluation of Fresh Berries and Their Structural Parts

The proportion (%) of the structural fruit parts (peel, pulp, and seeds) in fresh berries was obtained by gravimetrical determination of each element’s fresh weight (fw) (Mettler-Toledo, Switzerland; precision ± 0.0001 g) for 100 randomly selected fruits. Seed absolute weight (g) was obtained as mean by weighing of 1000 seeds on a precision balance (Mettler-Toledo, Switzerland; precision ± 0.0001 g).

The moisture content of each fruit sample in the study (seeds, peel, and pulp) was determined by drying in a laboratory drying oven (Robotika, Velingrad, Bulgaria) at 103 ± 2 °C to the constant weight [25]. The results from the chemical analyses in the study were further re-calculated and presented on a dry weight (dw) basis.

In the first step of chemical analysis, the samples were analyzed in two structural forms, as seed samples and peel/pulp samples.

Cellulose content (crude fiber) in the seed and peel/pulp samples was determined by a slight modification of a method described earlier [26]. The hydrolysis of the plant material (1.0 g) was carried out with 16.5 mL 80% CH_3_COOH and 1.5 mL concentrated HNO_3_ for 1.5 h at 100 °C. The filtrated residue was dried at 103 ± 2 °C for 24 h, cooled in a dessicator, and weighed for the quantitative determination of cellulose. The results are presented on a dry weight basis.

Protein content in the seed and peel/pulp fractions was analyzed by the Kjeldahl method [25] using an UDK 152 unit (Velp Scientifica Srl, Usmate Velate, Italy). The conversion to protein content of the determined nitrogen content, present as ammonia in the digested sample, was by the multiplication factor of 6.25.

Oil content in the seed and in the peel/pulp parts was determined after extraction with *n*-hexane (Soxhlet, for 8 h), followed by evaporation of the solvent on a rotary vacuum evaporator (at 40 °C) and under a stream of nitrogen [27].

### 2.3. Determination of Fatty Acids in Seed and Peel Oils

In the second step of the analysis, the oils from the fruit seeds and peel were isolated separately and then analyzed for the composition and content of the fatty acids and tocopherols. The oil fractions from seeds and peel were obtained by extraction with *n*-hexane, as described above [27].

Fatty acids in the extracted seed and peel oils were determined after transmethylation with 2% H_2_SO_4_ in CH_3_OH at 50 °C [28,29]. The GC analysis was performed on a Hewlett Packard 5890A unit, with a flame ionization detector (FID) (Santa Clara, CA, USA) and a capillary Supelco 2560 column, 75 m × 0.25 mm × 18 μm (i.d.). The column temperature increase was from 130 °C (4 min) to 240 °C (5 min) at 15 °C/min; injector/detector temperatures were 250 °C; the flow rate of the carrier gas (hydrogen) was 0.8 mL/min; the split was 50:1. Fatty acid identification was completed by referring to the retention times of fatty acid methyl esters (FAME) in a standard mixture of 37 components (Supelco, Bellefonte, PA, USA).

### 2.4. Determination of Tocopherols in Seed and Peel Oils

Tocopherols were analyzed directly in the seed and peel oils, without saponification, by HPLC method. A 2% solution of the respective oil in *n*-hexane was prepared, and 20 μL was injected in the Merck-Hitachi unit (Merck, Darmstadt, Germany); column 250 mm × 4 mm Nucleosil Si 50-5; fluorescent detector Merck-Hitachi F-1000 (Merck, Darmstadt, Germany). The flow rate of the mobile phase (*n*-hexane: dioxan 96:4, *v*/*v*) was 1.0 mL/min; detector excitation was at 295 nm, emission at 330 nm. Tocopherol identification was based on comparison with reference standards (DL-*α*-, DL-β-, DL-*γ*- and DL-*δ*-tocopherols, 98% purity) [30].

### 2.5. Determination of Amino Acids in Seed Cakes

In the third step, the seed cakes remaining after seed oil extraction were analyzed for the composition and content of amino acids and mineral elements.

Amino acids were detemined after hydrolysis of the seed cake material and completed with 6 N HCl at 105 °C for 24 h in sealed glass ampules. Ampule content was then evaporated at 40–50 °C under vacuum, and the residue was dissolved in 20 mM HCl and filtered. The free amino acids resulting from protein hydrolysis were derivatized using the AccQ-Fluor kit, WATO52880 (Waters Corporation, Milford, MA, USA). The HPLC separation of the resulting AccQ-Fluor amino acid derivatives was performed on an Elite LaChrome (Hitachi, Tokyo, Japan) unit, with a reverse phase C18 AccQ-Tag (3.9 mm × 150 mm) column (at 37 °C). WATO52890 buffer (Waters Corporation, Milford, MA, USA) and 60% acetonitrile were the eluting phases; the injected sample volume was 20 µL, heated to 55 °C. The unit was equipped with a diode array detector (DAD) (Hitachi, Tokyo, Japan), and the detection was performed at the wavelength 254 nm.

### 2.6. Determination of Mineral Elements in Seed Cakes

Mineral elements in the seed cakes were determined after mineralization at 450 °C; the resultant ash was first dissolved in concentrated HCl and then in 0.1 mol/L HNO_3_ [31]. The atomic absorption spectrometry (AAS) was performed on a Perkin Elmer/HGA 500 instrument (Norwalk, CT, USA). The detection wavelengths were: Na, 589.6 nm; K, 766.5 nm; Mg, 285.2 nm; Ca, 317.0 nm; Zn, 213.9 nm; Cu, 324.7 nm; Fe, 238.3 nm; Mn, 257.6 nm; Pb, 283.3 nm; Cd, 228.8 nm; Cr, 357.9 nm. The elemental identification was completed by comparison with standard metal salt solutions, and the estimation of metal ion concentration by using calibration curves (built for 1 μg/mL standard salt solutions).

### 2.7. Determination of Volatiles in Fruit Concretes

Finally, the fruit concretes were analyzed. These were obtained from *P. alkekengi* peel and pulp as concentrated aromatic products, which are commonly used in perfumery and cosmetics.

In the obtainment of fruit concretes, the samples were subjected to a double extraction with n-hexane, at a temperature of 40 °C and a solid-to-solvent ratio of 1:10 (*w*/*v*). The duration of the first and second extraction was 60 min and 30 min, respectively. The extracts were then combined and concentrated on a rotary vacuum evaporator until complete solvent removal at a temperature 40 °C. The yield of peel and pulp concretes was determined gravimetrically (%, *w*/*w*) and calculated on a dry weight basis. The initial moisture content of the extracted plant materials was as follows: peel—53.85 ± 0.45% (PA-SB) and 49.73 ± 0.41% (PA-NB); pulp—79.43 ± 0.62% (PA-SB) and 82.99 ± 0.71% (PA-NB). The color and appearance of the concretes were determined by visual assessment.

The GC analysis for the determination of the volatile composition of the obtained fruit concretes was performed on an Agilent 7890A instrument (Agilent Technologies Inc., Santa Clara, CA, USA), with the following parameters: HP-5ms column, 30 m × 250 mm × 0.25 µm (i.d.); oven temperature increased at a rate of 5 °C/min from 35 °C (3 min) to 250 °C (3 min), total run time 49 min; carrier gas (helium) at 1 mL/min constant rate; 30:1 split mode. The GC-MS analysis employed an Agilent 5975C inert XL EI/CI mass selective detector (MSD) (Agilent Technologies Inc., Santa Clara, CA, USA), under the same operational conditions as in the GC analysis. Mass spectra acquisition was at 70 eV in electron impact (EI) mode; MS scan was from 50 to 550 *m*/*z*. The ionization source temperature was 230 °C, and the MS quad and the injector temperatures were 150 °C and 250 °C, respectively. Mass spectra were read using the built-in toolkit of 2.64 AMDIS (Automated Mass Spectral Deconvolution and Identification System; NIST, Gaithersburg, MD, USA) software. The identification of volatiles was based on comparison of their retention (Kovats) indices (RI) and MS fragmentation patterns with spectral library data [32,33]. Components were listed in ascending order of the RI, calculated using a standard calibration mixture of *n*-alkanes (C_8_–C_40_) in hexane, under the same operational conditions. Compound concentrations were calculated as percentage of the total ion current (TIC), after normalization of the recorded peak areas.

### 2.8. Statistics

All measurements in the study were performed in triplicate (*n* = 3), except for the fruit parts proportion (*n* = 100). The results are presented as the mean value with the corresponding standard deviation (SD). ANOVA and Tukey multiple comparison test were used as statistical tools in the assessment of significant differences at *p* < 0.05.

## 3. Results and Discussion

### 3.1. Basic Evaluation of Fruit Structural Parts

The proportions of fruit structural parts (peel, seeds, and pulp, respectively) in the analyzed fresh fruits of two *P. alkekengi* phenotypes (denoted as PA-SB and PA-NB) are presented in Table 1. Fruit pulp accounted for about 70% of fresh fruit weight, while seeds constituted about a quarter of the fruit weight, with no significant deviations between the phenotypes. Seed dimensions varied, however, with seed absolute weight being 1.68 ± 0.01 g (mean of 1000 seeds results) for PA-SB phenotype, and 1.52 ± 0.01 g (per 1000 seeds) for PA-NB. The average number of seeds in a single berry was 195 ± 1.80 for PA-SB, and 107 ± 0.90 for PA-NB phenotype.

The basic macro component characteristics (cellulose, protein, oil) of the studied fruit fractions (the isolated seeds and the combined peel/pulp fraction) are presented in Table 2. The data indicate some differences between the phenotypes, with slightly higher oil amounts in PA-NB, and higher cellulose and protein amounts in the PA-SB phenotype. As seen from the data, the seeds were the primary site of oil accumulation in the fruit, although the combined peel/pulp samples also had detectable amounts of the oil fraction. The seeds of *P. alkekengi* were a sufficiently rich source of oil, yielding 14–17% oil, thus approximating the data for a related *Physalis* species, *P. peruviana* pomace oil (19.3%) detected by Ramadan [34], as well as those for soybean oil (18%) [35], grapeseed oil (8–20%) [36], or *P. alkekengi* seed oil [37].

Similarly, the seeds contained most of the fruit’s protein and fiber. The cellulose content was several times higher in the seeds (6–8% dw) than in the peel/pulp residues (1.5–2% dw). The reported data were close to the cellulose contents detected in the whole fruits of different cape gooseberry (*P. peruviana*) phenotypes measured by Petkova et al. [38], as well as in different fruit and vegetable pomaces, such as apples and tomatoes [39].

### 3.2. Determination of Fatty Acids in Seed and Peel Oils

The significant oil yield from the seeds of *P. alkekengi* fruit was the reason for subjecting the extracted seed oil to a more detailed analysis in order to reveal its micro component characteristics.

As seen in Table 2, the combined peel/pulp fraction was also associated with the presence of oil fraction, although in a minor amount. The individual analysis of the two fruit parts constituting the combined sample revealed that oil content in the pulp was minimal (below 1% dw) in both phenotypes, while the peel contained 2.54 ± 0.02% and 2.05 ± 0.02% oil (dw) in the PA-NB and PA-SB phenotypes, respectively. Therefore, it was considered interesting to identify the composition of the peel fraction oil, as well, in view of a more complete insight into the composition of *P. alkekengi* fruits.

**Figure 2:**
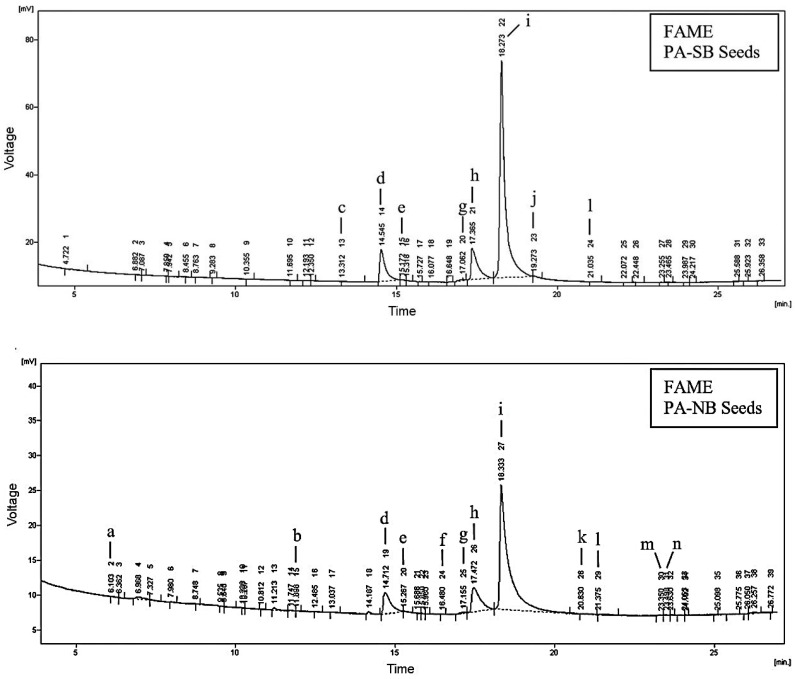
Chromatograms of the fatty acid composition of seed oils of two *P. alkekengi* phenotypes (PA-SB and PA-NB): a—capric acid; b—myristic acid; c—pentadecylic acid; d—palmitic acid; e—palmitoleic acid; f—heptadecenoic acid; g—stearic acid; h—oleic acid; i—linoleic acid; j—linolenic acid; k—eicosadienoic acid; l—eicosatrienoic acid; m—eicosapentaenoic acid; n—eicosatetraenoic acid.

The results regarding the fatty acid composition of the seed and peel oils of the two phenotypes are presented in Table 3, and an example of the obtained FAME chromatograms is shown in Figure 2. The data proved significant variations in the number and distribution of the identified fatty acids in the seed and peel oils, while the differences between the phenotypes were less pronounced.

The fatty acid composition of *P. alkekengi* seed oil was dominated by unsaturated fatty acids in a ratio of about 7:1 to saturated ones; this was the same for both phenotypes. The ratio between polyunsaturated and monounsaturated fatty acids was also favorable and comparable for the phenotypes, being about 5:1. The proportions of unsaturated and saturated fatty acids, however, were reversed and much more unfavorable in the peel oils, being 32:68 in PA-SB and 44:56 in PA-NB, respectively.

The predominant fatty acid in the seed oils was linoleic acid (73.67% in PA-SB and 74.43% in PA-NB), followed by oleic and palmitic acids, while the major fatty acids in peel oils were palmitic (57.88% in PA-SB and 36.21% in PA-NB) and oleic (24.02% in PA-SB and 30.08% in PA-NB) acids.

Unlike the similar distribution of fatty acids in the seed oils, there were differences between the two phenotypes in the individual fatty acid composition of the oil derived from fruit peel alone, especially with regard to palmitic, palmitoleic, stearic, and some other acids. Although the results revealed a more favorable composition in the isolated seed oils, they supported the feasibility of oil extraction from *P. alkekengi* fruit, using both whole fruit and seeds alone. Regarding the individual fatty acid composition of the seed oils, our results differed only numerically from the data in the previous study [37], which identified linoleic (86.9%) and palmitic (6.6%) acids as the major fatty acids in the seed oil, at a ratio of unsaturated to saturated fatty acids of about 14:1. Our results were fully compliant with the data available for *P. peruviana* oils [34], which pointed out that pulp/peel oil was characterized by high amounts of saturated fatty acids, while *ω*-3 acids (α-linolenic) were found in lower levels.

### 3.3. Determination of Tocopherols in Seed and Peel Oils

The tocopherol composition of the extracted oils is presented in Table 4. As seen in the table, there was impressive differentiation in the content of the bioactive tocopherols on the bases of fruit structural parts and phenotype. Seed oils, despite the phenotype-related differences observed, contained considerably more tocopherols than peel oils. The data showed about a 2.5 times higher concentration of the biologically active tocopherols in the oil isolated from the seeds of PA-SB berries than for the PA-NB phenotype.

The tocopherol fraction of both seed oils was predominated by *β*-tocopherol (70.63%, and 76.61%, of the total tocopherols, PA-SB and PA-NB, respectively), and *γ*-tocopherol (28.44% and 23.42% of the total content, PA-SB and PA-NB, respectively). The peel oils showed completely different tocopherol profiles—*γ*-tocopherol was dominating in PA-SB (69.74%), followed by *β*-tocopherol (30.32%), while *α*-tocopherol was detected as a single representative (100%) in PA-NB peel oil.

In the study of Ramadan [34], *β*- and *γ*-tocopherols were also the major tocopherols in the seed oil of *P. peruviana*, while *γ*- and *α*-tocopherols were the main components in the pulp/peel oil. No further parallel to other data could be made concerning the peel oil composition or the tocopherols, as to the best of our knowledge, there have been no other previous investigations in that direction.

### 3.4. Determination of Amino Acids in Seed Cakes

Accounting for the significant ratio of seeds in fresh fruit weight (Table 1) and their macronutrient indices (Table 2), the seed cakes resulting from oil extraction, otherwise considered a waste product, were considered a potentially valuable plant material worthy of recovery. Therefore, an attempt to provide new data in favor of their potential use and nutritional value was made in this study, by identifying some individual micro components in the seed cakes—amino acids and minerals. The results of the amino acid profile of seed cake proteins are presented in Table 5.

As seen from the data, the dominant amino acids in the seed cakes of both the *P. alkekengi* phenotypes were arginine, aspartic acid, glycine, and alanine, with numerical deviations between the samples. Respectively, there were differences in the distributions of the rest of the amino acids, explicable by the varying conditions of the plant vegetation (phenotype). The ratio of essential amino acids (EAA) was relatively low and comparable in the two phenotypes, being about 0.4:1. Valine, phenylalanine, threonine, and isoleucine were present in slightly higher amounts than the other EAAs.

### 3.5. Determination of Mineral Elements in Seed Cakes

The results from the determination of 11 key macro and micro mineral elements in the seed cakes (Table 6) confirmed significant differences between the phenotypes only with regard to two of the macro minerals, sodium and calcium; the content of sodium was substantially higher in the PA-SB phenotype, and that of calcium was higher in the seed cakes of PA-NB fruit. The macrominerals potassium and magnesium were found in identical amounts in both phenotypes. The identified microminerals (Fe, Mn, Cu, Zn, Pb) showed comparable distributions between the phenotypes, with the single exception of Cr in PA-NB.

Evaluating the potential of the seed cakes for nutritional purposes, they could be considered a good source of potassium (supplying about 15% of the reference dietary intake (RDI) for men and about 12% for women; the RDIs being 2700 and 3500 mg, respectively), magnesium (about 60% of RDI for men and about 70% of RDI for women; 350 and 300 mg), as well as of iron (about 60% of RDI for men and about 30% of RDI for women; 8 and 18 mg), zinc (about 20% of RDI for men and about 30% of RDI for women; 11 and 8 mg), and other key microelements (copper and manganese) [40].

### 3.6. Determination of Volatile Components in Fruit Concretes

Another important aspect of the study was the objective of investigating the potential of *P. alkekengi* fruit for obtaining fruit concrete, a type of established aromatic product, widely used in perfumery and cosmetics [41]. Concretes are obtained by extracting fresh or dry plant materials with a non-polar solvent (e.g., hexane, petroleum ether, or benzene), followed by the complete removal of the solvent; the resulting concentrated aromatic product carries a specific fragrance profile, adding to the diversification of the fragrance nuances provided by the other types of aromatic products from the respective plant material, such as essential oils, absolute, resinoids, tinctures, and other extracts [41].

Accounting for the fact that the fruit pulp and peel are generally the vegetal material strongly related to fruit flavor, those parts of the *P. alkekengi* fruit were processed individually by *n*-hexane double extraction to obtain the respective fruit concretes. All concretes obtained in the study represented thick waxy masses with dark orange–yellow color.

The results revealed significant differences in the yield of fruit concretes, both between the phenotypes and between the fruit structural parts compared in the study. As seen from the data in Table 7, the yield of peel concrete was significantly higher than that of pulp concrete, thus clearly differentiating the potential of the two fruit structures with regard to their processing efficiency. In turn, there were significant differences in peel concrete yield on a phenotype basis; it was about three time higher in PA-NB phenotype, which was obviously related to the impact of environmental characteristics on plant metabolism.

**Figure 3:**
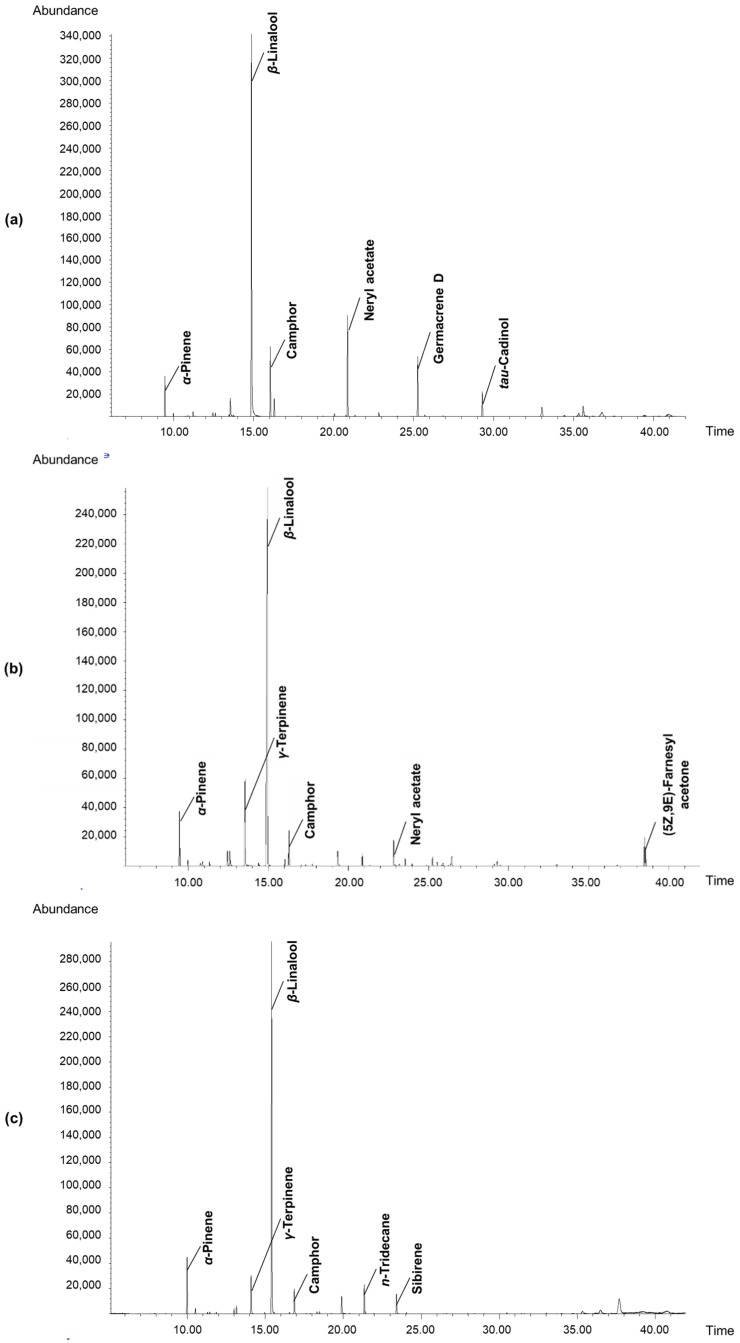

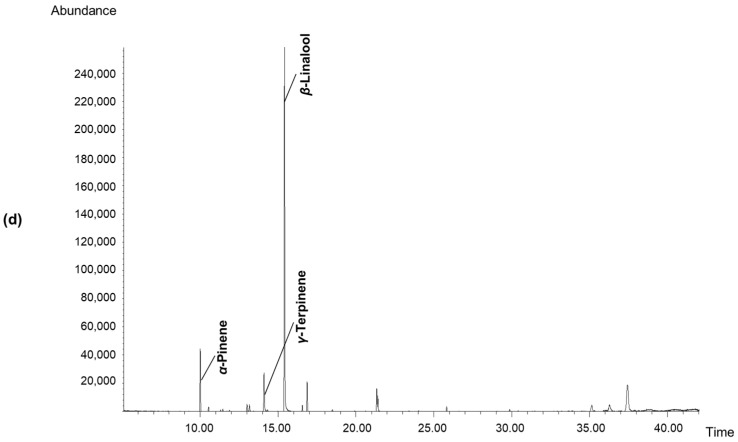
Chromatograms of volatiles in concretes obtained from two phenotypes of *P. alkekengi* fruit (PA-SB and PA-NB): (**a**) concrete from the peel of PA-SB phenotype; (**b**) concrete from the pulp of PA-SB phenotype; (**c**) concrete from the peel of PA-NB phenotype; (**d**) concrete from the pulp of PA-NB phenotype.

The results from the identification by GC-MS analysis of individual aromatic volatiles in the obtained concretes and the total ion current (TIC) chromatograms with the major compounds (over 3%) are presented in Table 8 and Figure 3, respectively.

Fifteen individual volatiles were identified by the applied GC-MS analysis in the pulp concrete of the PA-NB phenotype, accounting for 98.62% of the total content. The major components (in amounts over 3%) were: *β*-linalool (78.3%), *α*-pinene (6.9%), and *γ*-terpinene (5.5%). In the pulp concrete from the second phenotype, PA-SB, the number of identified compounds was 27 (98.56% of the total content), among which the major components were: *β*-linalool (61.9%), *γ*-terpinene (7.8%), α-pinene (5.3%), (5Z,9E)-farnesyl acetone (3.9%), neryl acetate (3.7%), and camphor (3.3%). The concrete obtained from the peel of the PA-NB phenotype contained 18 identified components (representing 98.41% of the total content). The major constituents in the product (over 3%) were: *β*-linalool (57.8%), *α*-pinene (7.6%), *n*-tridecane (6.2%), sibirene (5.9%), camphor (5.5%), and *γ*-terpinene (5.5%). In turn, a total of 23 individual components were identified in the peel concrete from the PA-SB phenotype (98.72% of the content), with the major constituents being: *β*-linalool (49.6%), neryl acetate (11.4%), camphor (7.5%), germacrene D (6.9%), *α*-pinene (4.6%), and *tau*-cadinol (3.7%).

The comparison of the data obtained for the respective aromatic products from two phenotypes revealed significant differences in the contents of a number of minor and major components with known fragrance properties; for instance, components in the pulp concretes included nerol (1.7% in PA-SB and 0.1% in PA-NB), neryl acetate (3.7% in PA-SB, not detected in PA-NB), and (5Z,9E)-farnesyl acetone (3.9% in PA-SB, not detected in PA-NB); components in the peel concretes included neryl acetate (11.4% in PA-SB and only 1.1% in PA-NB), sibirene (5.9% in PA-NB, not detected in PA-SB), germacrene D (6.9% in PA-SB and only 0.2% in PA-NB), and *tau*-cadinol (3.7% in PA-SB, not detected in PA-NB).

**Figure 4:**
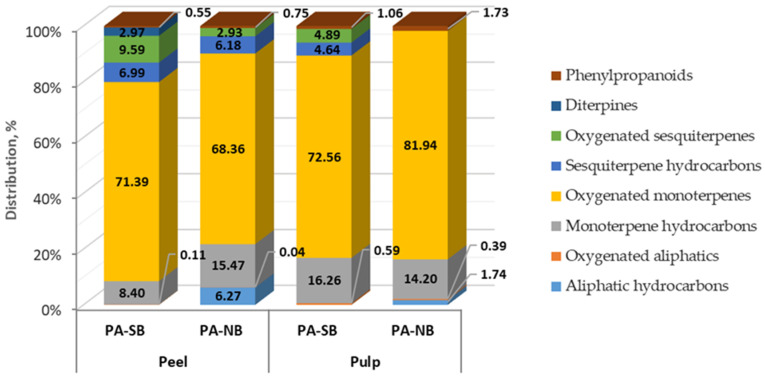
Distribution of volatiles by chemical groups in concretes obtained from two phenotypes of *P. alkekengi* fruit (PA-SB and PA-NB).

The classification of the identified fruit concrete constituents revealed the presence of compounds belonging to different chemical classes (Figure 4). The distribution of the identified compounds (equaled to 100%) did not suggest considerable differences between the two structural elements of the fruit (pulp, peel), or between the phenotypes, although some specifics also existed. In all extracts, the volatile composition was dominated by oxygenated monoterpene derivatives (72.6% and 81.9% in the pulp and 71.4% and 68.4% in the peel for the PA-SB and PA-NB phenotypes, respectively), followed by monoterpene hydrocarbons (16.3% and 14.2% in fruit pulp; 8.4% and 15.5% in the peel). Sesquiterpene hydrocarbons and oxygenated sesquiterpene derivatives were not detected in the concrete from the pulp of PA-NB phenotype, while diterpene representatives were found only in the concrete from the peel of PA-SB phenotype (3.0% of the identified content). The results revealed that the differences between the two phenotypes were more pronounced with regard to the extractible aromatic compounds in the peel compared with the fruit pulp. Thus, it could be presumed that fruit origin (phenotype) would not be a decisive factor for the organoleptic properties of the final product in *P. alkekengi* juice/pulp production, but would probably affect the usability of the resultant waste (peel or seed/peel residue); of course, additional and more targeted investigations are needed to support such assumptions, utilizing a wider range of sampling, parameter selection, and statistical tools.

## 4. Conclusions

The results achieved by this study conducted on *P. alkekengi* fruit from Bulgaria add new details to the existing knowledge about the species. The study revealed that each of the fruit structural parts (peel, pulp, and seeds) or the resultant by-products (the seed cakes), rarely analyzed individually, had its specific features in terms of the assessed chemical characteristics, thus suggesting different options for their prospective use as sources of functional macro- and micronutrients—fiber, protein and some essential amino acids, oils and unsaturated fatty acids, tocopherols, and minerals. The study also provides new data on the obtaining and identification of the volatile profiles of *P. alkekengi* fruit concretes, thus contributing to the expansion of the range of available aromatic products. The presence of a number of aroma-active volatile compounds in the obtained peel and pulp concretes spoke in favor of a tangible potential for their future consideration as ingredients, e.g., in perfumery and cosmetic formulations. The results on the phenotype-related differences presented in the study also suggested that fruit origin could, to a lesser or greater extent, affect the chemical composition of the assessed individual parts of *P. alkekengi* fruit and the aromatic products derived from them, especially if considering fruit properties on a wider geographical basis, in which genotype would most probably be another decisive factor. Based on these considerations, the outcomes from the study could be the grounds for future investigations in the indicated directions.

## Figures and Tables

**Table 1:** Proportion of fruit structural parts of two *P. alkekengi* phenotypes (PA-SB and PA-NB).

Fruit Part	PA-SB (% fw)	PA-NB (% fw)
Peel	4.99 ± 0.05 ^a^	5.43 ± 0.05 ^a^
Seeds	23.58 ± 0.18 ^b^	26.15 ± 0.23 ^b^
Pulp	71.43 ± 0.73 ^c^	68.42 ± 0.64 ^c^

Results: mean value ± standard deviation (*n* = 100). Different letters in the same row indicate significant differences (*p* ˂ 0.05).

**Table 2:** Macro component characteristics of fruit structural parts of two *P. alkekengi* phenotypes (PA-SB and PA-NB).

Component	PA-SB		PA-NB	
	Seeds (% dw)	Peel/Pulp (% dw)	Seeds (% dw)	Peel/Pulp (% dw)
Cellulose	8.06 ± 0.07 ^d^	1.44 ± 0.01 ^a^	6.12 ± 0.05 ^c^	2.15 ± 0.01 ^b^
Protein	19.14 ± 0.14 ^d^	2.51 ± 0.01 ^b^	16.22 ± 0.12 ^c^	1.94 ± 0.01 ^a^
Oil	14.13 ± 0.12 ^c^	1.27 ± 0.01 ^a^	17.57 ± 0.15 ^d^	1.81 ± 0.01 ^b^

Results: mean value ± standard deviation (*n* = 3). Different letters in the same row indicate significant differences (*p* ˂ 0.05).

**Table 3:** Fatty acid composition of seed and peel oils of two *P. alkekengi* phenotypes (PA-SB and PA-NB).

Fatty Acid		PA-SB		PA-NB	
		Seeds (% dw)	Peel (% dw)	Seeds (% dw)	Peel (% dw)
C_10:0_	Capric	nd ^1^	nd	0.10 ± 0.0 ^a^	0.09 ± 0.0 ^a^
C_11:0_	Undecylic	nd	0.33 ± 0.0 ^b^	nd	0.17 ± 0.0 ^a^
C_12:0_	Lauric	nd	1.08 ± 0.0 ^a^	nd	1.11 ± 0.01 ^a^
C_13:0_	Tridecylic	nd	0.09 ± 0.0 ^a^	nd	0.42 ± 0.0 ^b^
C_14:0_	Myristic	nd	3.89 ± 0.01 ^b^	0.11 ± 0.0 ^a^	5.01 ± 0.02 ^c^
C_14:1_	Myristoleic	nd	nd	nd	0.82 ± 0.0 ^a^
C_15:0_	Pentadecylic	0.21 ± 0.0 ^a^	nd	nd	4.44 ± 0.01 ^b^
C_16:0_	Palmitic	11.28 ± 0.10 ^b^	57.88 ± 0.42 ^d^	10.49 ± 0.10 ^a^	36.21 ± 0.29 ^c^
C_16:1_	Palmitoleic	0.12 ± 0.0 ^a^	1.79 ± 0.01 ^b^	0.18 ± 0.0 ^a^	8.22 ± 0.02 ^c^
C_17:0_	Margaric	nd	0.41 ± 0.0 ^a^	nd	0.57 ± 0.0 ^a^
C_17:1_	Heptadecenoic	nd	0.69 ± 0.0 ^b^	0.11 ± 0.0 ^a^	0.49 ± 0.0 ^b^
C_18:0_	Stearic	0.51 ± 0.0 ^a^	4.32 ± 0.02 ^b^	0.42 ± 0.0 ^a^	8.37 ± 0.02 ^c^
C_18:1_	Oleic	13.88 ± 0.11 ^a^	24.02 ± 0.31 ^b^	13.39 ± 0.12 ^a^	30.08 ± 0.21 ^c^
C_18:2_ (n-6)	Linoleic	73.67 ± 0.72 ^b^	1.00 ± 0.01 ^a^	74.43 ± 0.71 ^b^	0.83 ±0.0 ^a^
C_18:3_ (n-3)	Linolenic	0.21 ± 0.0 ^a^	2.38 ± 0.01 ^c^	nd	2.09 ± 0.02 ^b^
C_20:2_ (n-6)	Eicosadienoic	nd	2.12 ± 0.01 ^c^	0.28 ± 0.0 ^a^	1.08 ± 0.01 ^b^
C_20:3_ (n-6)	Eicosatrienoic	0.12 ± 0.0 ^a^	nd	0.29 ± 0.0 ^b^	nd
C_20:4_ (n-6)	Eicosatetraenoic	nd	nd	0.10 ± 0.0	nd
C_20:5_ (n-3)	Eicosapentaenoic	nd	nd	0.10 ± 0.0	nd
Saturated fatty acids		12.00	68.00	11.12	56.40
Unsaturated fatty acids, of which		88.00	32.00	88.88	43.60
Monounsaturated fatty acids		14.00	26.50	13.68	39.60
Polyunsaturated fatty acids		74.00	5.50	75.20	4.00

nd ^1^ = not detected; Results: mean value ± standard deviation (*n* = 3). Different letters in the same row indicate significant differences (*p* ˂ 0.05).

**Table 4:** Tocopherol composition of seed and peel oils of two *P. alkekengi* phenotypes (PA-SB and PA-NB).

Tocopherols	PA-SB		PS-NB	
	Seeds	Peel	Seeds	Peel
*α*-Tocopherol (% of the total tocopherols)	1.01 ± 0.01 ^a^	nd ^1^	nd	100 ± 0.01 ^b^
*β*-Tocopherol (% of the total tocopherols)	70.63 ± 0.68 ^b^	30.32 ± 0.20 ^a^	76.61 ± 0.71 ^c^	nd
*γ*-Tocopherol (% of the total tocopherols)	28.44 ± 0.22 ^b^	69.74 ± 0.31 ^c^	23.42 ± 0.21 ^a^	nd
Total tocopherols (mg/kg dw)	5378 ± 51.00 ^d^	340.00 ± 17.00 ^b^	2009 ± 20.00 ^c^	216.00 ± 11.00 ^a^

^1^ nd = not detected; Results: mean value ± standard deviation (*n* = 3); Different letters in the same row indicate significant differences (*p* ˂ 0.05).

**Table 5:** Amino acid composition of seed cakes from *P. alkekengi* fruit phenotypes (PA-SB and PA-NB).

Amino Acid	PA-SB (mg/g dw)	PA-NB (mg/g dw)
Aspartic acid	12.23 ± 0.11 ^a^	12.16 ± 0.11 ^a^
Serine	8.41 ± 0.07 ^b^	6.08 ± 0.06 ^a^
Glutamic acid	7.12 ± 0.07 ^a^	11.97 ± 0.10 ^b^
Glycine	10.94 ± 0.08 ^a^	14.83 ± 0.11 ^b^
Histidine	7.18 ± 0.07 ^b^	5.90 ± 0.05 ^a^
Arginine	15.75 ± 0.11 ^b^	13.17 ± 0.11 ^a^
Threonine ^1^	4.17 ± 0.03 ^b^	2.89 ± 0.03 ^a^
Alanine	9.31 ± 0.08 ^a^	14.34 ± 0.12 ^b^
Proline	2.54 ± 0.01 ^a^	4.27 ± 0.02 ^b^
Cysteine	0.24 ± 0.0 ^b^	0.14 ± 0.0 ^a^
Tyrosine	3.20 ± 0.02 ^a^	6.19 ± 0.04 ^b^
Valine ^1^	4.53 ± 0.02 ^b^	3.56 ± 0.02 ^a^
Methionine ^1^	0.77 ± 0.0 ^a^	0.70 ± 0.0 ^a^
Lysine ^1^	2.94 ± 0.01 ^a^	3.49 ± 0.02 ^b^
Isoleucine ^1^	3.37 ± 0.01 ^a^	4.41 ± 0.01 ^b^
Leucine ^1^	0.56 ± 0.0 ^a^	0.86 ± 0.0 ^b^
Phenylalanine ^1^	3.19 ± 0.02 ^a^	4.63 ± 0.02 ^b^

Results: mean value ± standard deviation (*n* = 3). Different letters in the same row indicate significant differences (*p* ˂ 0.05). ^1^ essential amino acid.

**Table 6:** Mineral composition of seed cakes from *P. alkekengi* fruit of two phenotypes (PA-SB and PA-NB).

Mineral Element	PA-SB (mg/kg dw)	PA-NB (mg/kg dw)
Potassium (K)	4122.28 ± 19.43 ^a^	4668.32 ± 21.23 ^a^
Sodium (Na)	182.33 ± 1.33 ^b^	80.79 ± 0.33 ^a^
Calcium (Ca)	529.15 ± 1.87 ^a^	1586.23 ± 4.78 ^b^
Magnesium (Mg)	2418.97 ± 11.24 ^a^	2318.26 ± 11.09 ^a^
Iron (Fe)	61.71 ± 0.21 ^b^	50.38 ± 0.19 ^a^
Manganese (Mn)	25.40 ± 0.09 ^a^	23.42 ± 0.08 ^a^
Copper (Cu)	9.98 ± 0.03 ^a^	10.30 ± 0.04 ^a^
Zinc (Zn)	29.64 ± 0.09 ^b^	25.24 ± 0.08 ^a^
Lead (Pb)	2.67 ± 0.0 ^a^	2.93 ± 0.0 ^a^
Cadmium (Cd)	nd ^1^	nd
Chromium (Cr)	nd	2.32 ± 0.0

Results: mean value ± standard deviation (*n* = 3). Different letters in the same row indicate significant differences (*p* ˂ 0.05). ^1^ nd = not detected.

**Table 7:** Primary characteristics of fruit concretes obtained from two phenotypes of *P. alkekengi* (PA-SB and PA-NB).

Index	PA-SB (% DW; *w*/*w*)	PS-NB (% DW; *w*/*w*)
Yield of pulp concrete	0.02 ± 0.00 ^a^	0.03 ± 0.00 ^a^
Yield of peel concrete	0.72 ± 0.01 ^a^	2.16 ± 0.01 ^b^

Results: mean value ± standard deviation (*n* = 3). Different letters in the same row indicate significant differences (*p* ˂ 0.05).

**Table 8:** Volatile components obtained by GC-MS in the concretes from two phenotypes of *P. alkekengi* (PA-SB and PA-NB) fruit.

Volatiles	RT ^1^	RI ^2^	PA-SB (% or TIC ^2^)		PA-NB (% or TIC ^3^)	
			Peel	Pulp	Peel	Pulp
*α*-Pinene	9.45	933	4.63 ± 0.03 ^a^	5.30 ± 0.04 ^b^	7.57 ± 0.06 ^d^	6.85 ± 0.05 ^c^
Camphene	9.98	945	0.31 ± 0.0 ^a^	0.53 ± 0.0 ^b^	0.69 ± 0.0 ^c^	0.28 ± 0.0 ^a^
Sabinene	10.76	969	0.05 ± 0.0 ^a^	0.22 ± 0.0 ^b^	0.08 ± 0.0 ^a^	0.15 ± 0.0 ^b^
*β*-Pinene	10.90	975	0.09 ± 0.0 ^a^	0.41 ± 0.0 ^c^	0.06 ± 0.0 ^a^	0.12 ± 0.0 ^b^
Myrcene	11.33	987	0.44 ± 0.0 ^b^	0.59 ± 0.0 ^c^	0.23 ± 0.0 ^a^	0.25 ± 0.0 ^a^
*p*-Cymene	12.45	1020	0.54 ± 0.0 ^a^	1.04 ± 0.09 ^c^	0.74 ± 0.0 ^b^	0.56 ± 0.0 ^a^
Limonene	12.60	1023	0.43 ± 0.0 ^a^	1.21 ± 0.03 ^d^	1.07 ± 0.09 ^c^	0.89 ± 0.0 ^b^
*γ*-Terpinene	13.54	1055	2.34 ± 0.01 ^a^	7.77 ± 0.06 ^c^	5.52 ± 0.05 ^b^	5.46 ± 0.05 ^b^
Camphenilone	14.40	1077	0.35 ± 0.0 ^b^	0.41 ± 0.0 ^b^	0.12 ± 0.0 ^a^	0.09 ± 0.0 ^a^
*β*-Linalool	14.92	1095	49.57 ± 0.47 ^a^	61.91 ± 0.60 ^c^	57.76 ± 0.50 ^b^	78.29 ± 0.70 ^d^
Nonanal	15.02	1101	0.11 ± 0.0 ^b^	0.58 ± 0.0 ^d^	0.04 ± 0.0 ^a^	0.38 ± 0.0 ^c^
Camphor	16.29	1140	7.53 ± 0.06 ^d^	3.26 ± 0.03 ^b^	5.53 ± 0.05 ^c^	2.31 ± 0.02 ^a^
1-Terpinen-4-ol	17.31	1174	0.04 ± 0.0 ^a^	0.18 ± 0.0 ^b^	nd ^4^	nd
*α*-Terpineol	17.75	1185	0.09 ± 0.0 ^a^	0.32 ± 0.0 ^b^	nd	nd
Nerol	19.33	1126	1.48 ± 0.01 ^b^	1.73 ± 0.01 ^c^	2.80 ± 0.02 ^d^	0.06 ± 0.0 ^a^
*n*-Tridecane	21.34	1300	nd	nd	6.18 ± 0.05 ^b^	1.72 ± 0.01 ^a^
Isoamyl benzyl ether	21.40	1310	nd	nd	nd	1.15 ± 0.01
Neryl acetate	22.83	1349	11.42 ± 0.10 ^c^	3.71 ± 0.03 ^b^	1.06 ± 0.01 ^a^	nd
Sibirene	23.55	1398	nd	0.72 ± 0.0 ^a^	5.85 ± 0.05 ^b^	nd
*β*-Caryophyllene	23.98	1419	nd	0.33 ± 0.0	nd	nd
Germacrene D	25.24	1483	6.85 ± 0.06 ^c^	0.88 ± 0.0 ^b^	0.23 ± 0.03 ^a^	nd
*β*-Selinene	25.40	1491	0.05 ± 0.0 ^a^	0.43 ± 0.0 ^b^	nd	nd
*α*-Zingiberene	25.61	1493	nd	0.27 ± 0.0	nd	nd
Bicyclogermacrene	25.91	1501	nd	0.55 ± 0.0	nd	nd
*δ*-Cadinene	26.55	1522	nd	1.07 ± 0.01	nd	nd
*α*-Cadinene	26.80	1536	nd	0.32 ± 0.05	nd	nd
1-epi-Cubenol	29.11	1627	nd	0.41 ± 0.05	nd	nd
*tau*-Cadinol	29.30	1640	3.69 ± 0.03 ^b^	0.54 ± 0.0 ^a^	nd	nd
(2E,6E)-Methyl farnesoate	35.12	1785	1.11 ± 0.01	nd	nd	nd
(2Z,6E)-Farnesyl acetate	36.19	1820	2.58 ± 0.02	nd	nd	nd
(5Z,9E)-Farnesyl acetone	38.30	1889	2.09 ± 0.02 ^b^	3.87 ± 0.03 ^c^	2.88 ± 0.02 ^a^	nd
Phytol	40.74	1940	2.93 ± 0.02	nd	nd	nd

^1^ RT—retention time, min; ^2^ RI—retention index (Kovat’s index); ^3^ TIC—total ion current; Results: mean value ± standard deviation (*n* = 3). Different letters in the same row indicate significant differences (*p* ˂ 0.05). ^4^ nd = not detected.

## Data Availability

The data presented in this study are available on request from the authors.
